# Restoration of tryptophan hydroxylase functions and serotonin content in the Atlantic croaker hypothalamus by antioxidant treatment during hypoxic stress

**DOI:** 10.3389/fnins.2014.00130

**Published:** 2014-05-30

**Authors:** Md. Saydur Rahman, Peter Thomas

**Affiliations:** Marine Science Institute, University of Texas at AustinPort Aransas, TX, USA

**Keywords:** neuroenzyme, neurotransmitter, antioxidant, fish, brain, hypoxia

## Abstract

Antioxidants are prototypical scavengers of oxygen-free radicals and have been shown to prevent neuroendocrine dysfunction in vertebrates during oxidative stress. In the present study, we investigated whether antioxidant treatment can reverse hypoxia-induced down-regulation of hypothalamic tryptophan hydroxylase (TPH) and serotonergic functions in Atlantic croaker. Hypothalamic neuronal contents of TPH-1 and TPH-2 proteins, serotonin (5-hydroxytryptamine, 5-HT) and its precursor, 5-hydroxytryptophan (5-HTP) as well as hypothalamic TPH-1 and TPH-2 mRNA expression and TPH activity were measured in croaker after exposure to hypoxia and treatment with pharmacological agents. Multiple injections of *N*-ethylmaleimide, a sulfhydryl alkylating agent, caused comparable decreases in hypothalamic TPHs functions and 5-HT contents to that induced by hypoxia exposure (dissolved oxygen: 1.7 mg/L for 4 weeks) which were partially restored by repeated injections with a nitric oxide synthase (NOS)-inhibitor and/or vitamin E. Double-labeled immunohistochemical results showed that TPHs and 5-HT neurons were co-expressed with neuronal NOS (nNOS, a neuroenzyme) that catalyzes the production of nitric oxide, a free radical, in hypothalamic neurons. These results suggest that hypoxia-induced impairment of TPH and serotonergic functions are mediated by nNOS and involve the generation of free radicals and a decrease in the antioxidant status. This study provides, to our knowledge, the first evidence of a protective role for an antioxidant in maintaining neural TPHs functions and 5-HT regulation in an aquatic vertebrate during hypoxic stress.

## Introduction

The brains of vertebrates are particularly susceptible to decreases in oxygen levels (Lahiri et al., 2006). A deficiency of oxygen and/or antioxidant status often results in neural dysfunction in vertebrates (Zingg and Azzi, 2004; Lahiri et al., 2006; Traber and Stevens, 2011). Impairment of neural functions often occurs when aquatic vertebrates, particularly fish, are frequently exposed to low oxygen conditions in their environment (Thomas and Rahman, 2009; Wu, 2009). The neurons that synthesize the neurotransmitter serotonin (5-HT) appear to be especially sensitive to low oxygen levels. Tryptophan hydroxylase (TPH) is the rate-limiting enzyme in 5-HT synthesis and minor changes in TPH activity can cause drastic changes in 5-HT content and serotonergic functions. TPH is an oxygen-liable neuroenzyme, and maintenance of adequate oxygen levels is essential for maintaining its enzymatic activity (Kuhn et al., 1980). Therefore, the extreme sensitivity of serotonergic functions to alterations in neuronal levels of oxygen are largely due to this requirement of TPH (Roberts and Fitzpatrick, 2013). The serotonergic system in teleosts differs from that in mammals because the 5-HT neurons controlling reproductive neuroendocrine functions are mainly localized in the hypothalamic region, separate from those controlling other neuronal functions of 5-HT in the CNS (Jacobs and Azmitia, 1992; Khan and Thomas, 1993; Mohammad-Zadeh and Gwaltney-Brant, 2008). Thus, the teleost hypothalamus is an excellent vertebrate model for examining the effects of hypoxia on serotonergic functions specifically controlling reproductive neuroendocrine functions and the molecular mechanisms involved.

Hypoxia (dissolved oxygen concentration <2.0 mg/L defined as hypoxia, Diaz and Rosenberg, 2008) is a severe environmental stress that has pronounced effects on reproduction by impairing gonadal development and gonadotropin secretion from the pituitary (Thomas et al., 2007; Wu, 2009; Thomas and Rahman, 2012). Hypoxia also disrupts brain functions by decreasing neuropeptide and neurotransmitter levels and neuroenzyme activities (Hedner and Lundborg, 1979; Thomas et al., 2007; Wu, 2009; Gilany and Vafakhah, 2010; Kumar, 2011). In recent *in vivo* studies, we have shown that hypoxia markedly decreases TPHs (TPH isoforms: TPH-1 and TPH-2) immunoreactive (IR) neuronal expression, mRNA and protein levels, and TPH activity in the hypothalamus of Atlantic croaker, a relatively hypoxia-tolerant marine fish (Rahman and Thomas, 2009). We have also shown that these declines in TPH expression are accompanied by decreases in hypothalamic 5-hydroxytryptophan (5-HTP, an immediate precursor of 5-HT) and 5-HT contents, and gonadotropin-releasing hormone-I (GnRH-I) mRNA levels in croaker hypothalamus (Thomas et al., 2007; Rahman et al., 2011). These hypoxia-induced neuroendocrine dysfunctions also lead to decreased pituitary luteinizing hormone secretion and plasma sex steroid hormone levels, resulting in impairment of gonadal development and reproductive success in croaker and other teleost fishes (Thomas et al., 2007; Wu, 2009). Experimental *in vivo* studies in tetrapods have also shown that hypoxia decreases TPH activity and 5-HT contents in neonatal and adult rat brains (Davis et al., 1973; Hedner and Lundborg, 1979; Poncet et al., 1997). Collectively, these studies indicate that serotonergic transmission in vertebrates is extremely susceptible to disturbance by hypoxia.

Antioxidants such as vitamin A, C, and E, are essential nutrients necessary for optimal growth, development, and reproduction in animals (Evans and Bishop, 1922; Traber and Stevens, 2011). Among them, vitamin E (Vit E) is a potent antioxidant which regulates neuronal function(s) and maintains cellular integrity (Muller, 2010). Vit E also plays an important role in cell signaling (Azzi, 2007). Mounting evidence has also accumulated that Vit E exerts protective effects against oxidative stress and prevents the propagation of reactive oxygen species (ROS, such as superoxide anion, O^−^_2_; hydroxyl radical, OH·) and reactive nitrogen species (RNS, such as nitric oxide, NO; peroxynitrate, ONOO^−^) (Chow, 1991; Chow et al., 1999; Traber and Stevens, 2011). Numerous studies have reported that hypoxia increases cellular O^−^_2_ and NO levels which induce oxidative stress, leading to increased neuronal apoptosis and necrosis (Cazevieille et al., 1993; Tagami et al., 1998; Yamagata et al., 2010). Subsequently, overproduced of O^−^_2_ and NO rapidly react with each other to generate ONOO^−^, a highly reactive molecule, which attacks neurons, cells, and tissues as well as depleting antioxidant enzymes activities (Freidovich, 1999; Kelm, 1999; Lièvre et al., 2000). Several lines of evidence indicate that NO and ONOO^−^ directly inactivate the enzymatic activity of TPH and exposure of this enzyme to oxidizing conditions rapidly destroys its catalytic function (Kuhn and Arthur, 1996, 1997a,b; Kuhn and Geddes, 1999, 2000). Therefore, we hypothesize that under hypoxic conditions when large amounts and varieties of radicals are produced, administration of Vit E may restore TPH activity as well as serotonergic functions in the vertebrate brain.

The aims of the present study were to investigate whether treatment with Vit E reverses the inhibitory effects of hypoxia on TPH levels and TPH activity and 5-HT content in the hypothalamic tissues of Atlantic croaker. We investigated the role of nitric oxide synthase (NOS, an enzyme) in the hypoxia-induced effects on hypothalamic serotonergic function by investigating whether TPH and 5-HT expression could be restored by systemic administration of a NOS-inhibitor. Finally, to evaluate the potential role of alterations sulfhydryl (SH) groups on TPH in hypoxia impairment of serotonergic functions, we tested whether the hypoxia effects are mimicked by treatment with a SH alkylating agent.

## Materials and methods

### Chemicals

L-[5-^3^H]-tryptophan citation (27 Ci/mmol) and 5-hydroxytryptamine (5-HT, serotonin) were purchased from Amersham Biosciences (Piscataway, NJ) and Sigma-Aldrich (St. Louis, MO), respectively. Rabbit polyclonal anti-TPH-1 and -TPH-2 antibodies and TPHs peptides were generous gifts from Dr. Donald M. Kuhn, Wayne State University School of Medicine, Detroit, MI and the specificity of both antibodies has been demonstrated previously (Sakowski et al., 2006). Commercially available antibodies, rabbit anti-5-HT, goat polyclonal to rabbit IgG (HRP), and rabbit anti-actin were purchased from ImmunoStar (Hudson, WI), SouthernBiotech (Birmingham, AL), and Novus Biologicals (Littleton, CO), respectively. Materials for molecular biology were obtained from Agilent Technologies (La Jolla, CA), Promega (Madison, WI), and Invitrogen (Carlsbad, CA). All other chemicals were purchased from Sigma-Aldrich and Fisher Scientific (Pittsburgh, PA).

### Experimental fish

Juvenile (year 1) Atlantic croaker, *Micropogonias undulatus* (10–11 cm length; 25–35 g body weight, BW), were purchased from local bait shops during the summer and transported to fish holding facilities at the University of Texas Marine Science Institute, Port Aransas, Texas. Fish were treated with Paracide-F (Argent Chemical, Redmond, WA) at 170 ppm in seawater to minimize parasite infections and transferred to large indoor recirculating seawater tanks (5000 L) and maintained under ambient temperature (22–23°C) and photoperiod (13D:11L) for 3 months by which time they had become sexually mature. Chopped shrimp were fed to the fish once a day (3% BW/day).

### Experiment: effects of hypoxia and pharmacological agents on TPH and 5-HT regulation

Sexually mature croakers were stocked into 12 tanks (30 mixed-sex fish/tank) with a recirculating seawater system (capacity 2025 l, including biological filter) under constant temperature/photoperiod conditions (22 ± 1°C, 13D:11L) for 1 month prior to experimentation. The hypoxia experimental setup used in this study has been described previously (Rahman and Thomas, 2009). Briefly, six tanks were maintained under normoxic conditions (dissolved oxygen, DO: 6.5 mg/L) and the other six tanks were assigned hypoxia treatments (1.7 mg DO/L). The DO levels in the hypoxia exposure tanks were lowered by reducing the aeration gradually with air regulators and adjusted until the DO level reached 1.7 mg/L which was achieved within 2 days. DO, pH, and temperature levels were measured with a YSI multiprobe meter (YSI 556 Multiprobe System, YSI Incorporated, Yellow Springs, OH) three times a day in the morning, afternoon and night. Ammonia and nitrite levels were monitored using Hach kits (HACH, Loveland, CO) three times a week. There were no major changes of the physio-chemical parameters (pH 7.7–7.9, ammonia 0.1–0.2 mg/L, and nitrite 0.01–0.02 mg/L) during the experimental periods.

Fish were anesthetized with quinaldine sulfate (20 mg/L), rapidly weighed and received intraperitoneal (i.p.) injections with vehicle, *N*-ethylmaleimide (NEM, a chemical which covalently modifies sulfhydryl groups in the reactive center of enzymes), *N*ω-nitro-L-arginine methyl ester (NAME, an inhibitor of nitric oxide synthase), or vitamin E (α-tocopherol, an antioxidant) every 4 days for 4 weeks (6 i.p. injections of 1 μg NEM, 1 μg NAME, or 1 μg Vit E/g BW). At the end of the experiments, the fish were sacrificed under deep anesthesia using quinaldine sulfate (20 mg/L), following guidelines and ethical rules approved by the University of Texas at Austin Animal Care and Use Committee (IACUC, protocol# 09022701). Brain tissues were quickly excised, frozen in liquid nitrogen and stored at −80°C. Hypothalamic tissues were excised from frozen brain samples with the aid of a croaker brain atlas (Khan and Thomas, 1993) for RNA extraction, protein determination, and neurotransmitter measurement. For radioenzymatic assay, hypothalamic tissue samples were separated from fresh brain samples. For immunohistochemical detection, whole brains were fixed in ice cold paraformaldehyde solution.

### Single- and double-immunofluorescence staining of TPHs and 5-HT neurons

Details the single-immufluorescence staining methods were used in this study have been described previously (Rahman and Thomas, 2009, 2013). Briefly, whole brains were stored in paraformaldehyde solution (4% paraformaldehyde in 0.01 M phosphate-buffered saline, PBS, pH 7.4) overnight at 4°C. The following day, brains were dehydrated in a series of ethanol solutions, embedded in paraffin (Paraplast, Sigma-Aldrich), and sectioned at 10 μm on a rotary microtome in transverse and sagittal planes. Sections were mounted on superfrost plus slides (Fisher Scientific), deparaffinized in xylene, dehydrated in a series of ethanol solutions, and rinsed with PBS. Endogenous peroxidase activity was blocked with 5% H_2_O_2_ for 10 min at room temperature. Sections were treated in retrieval solution (1 mM Tris, pH 9.0, with 0.5 mM EGTA) for 10 min to facilitate the immunoreactions. Nonspecific binding was prevented by blocking in PBS containing 1% BSA. The immunofluorescent signals of TPHs and 5-HT neurons in croaker hypothalamus were amplified by tyramide signal amplification solution (Molecular Probes, Eugene, OR) using a signal-labeling technique. Sections were incubated with TPH-1, TPH-2, or 5-HT antibodies at a dilution of 1:100 overnight at 4°C. For peptide block controls, each antigenic TPHs peptide was diluted in blocking buffer (400 ng/ml) containing it's respective antibody (dilution: 1:100) and preabsorbed overnight at 4°C. The fluorescence signal was amplified by adding Alexa Fluor 594 or Alexa Fluor 488 goat anti-rabbit secondary antibodies (dilution 1:500, Invitrogen) and incubating for 1 h in the dark. The sections were rinsed three times in PBS and mounted with Prolong Gold antifade reagent (Invitrogen). The presence of the fluorescent-labeled TPH-1, TPH-2 and 5-HT neurons was examined using a Nikon Eclipse E600 microscope (Nikon, Japan) with fluorescein red and green filters. The image was captured by Cool-SNAP camera (Photometrics, Tucson, AZ), and the intensity of each neuron was quantified by NIH ImageJ analysis software (http://rsb.info.nih.gov/ij/), a computer-assisted scientific image processing program (Schneider et al., 2012), according to Collins (2007).

A double-immunofluorescence method described by Kroeber et al. (1998) which employs two primary antibodies raised in the same species was used with minor modifications to detect neuronal NOS (nNOS) and TPHs or 5-HT in the same neurons. Briefly, rabbit polyclonal anti-nNOS antibody (SC-1025, Santa Cruz, CA) was purified by Melon Gel IgG purification column (catalog# 45206, Thermo Fisher Scientific, Rockford, lL). The IgG purified antibody was then directly labeled with DyLight-488 antibody (catalog# 53024, Thermo Scientific) according to the manufacturer's protocol. Sections were incubated with unlabeled rabbit TPH-1, TPH-2, or 5-HT primary antibodies (1:100) overnight at 4°C, rinsed three times with PBS and incubated with fluorescent Rhodamine Red-X conjugated goat anti-rabbit secondary antibody (1:500; Jackson Immunoresearch, West Grove, PA) for 1 h in the dark. Sections were rinsed with PBS, and incubated with 5% normal rabbit serum in PBS-T (PBS with 0.3% TritonX-100) to occupy any free binding site for rabbit antibody remaining on the secondary antibody. Sections were then blocked with blocking solution (3% normal goat serum and 0.3% TritonX-100 in PBS) for 1 h at room temperature, rinsed three times with PBS, and incubated with anti-nNOS-DyLight 488 labeled-antibody (1:500) at 4°C overnight in a humidified chamber the dark. Sections were then rinsed three times in PBS and mounted in Fluoromount-G solution (a non-fluorescing solution, SouthernBiotech). The presence of the double-labeled immunofluorescence signal was visualized using a confocal microscope (Nikon Eclipse C2, Nikon, Japan).

### Western blot analysis of TPHs proteins

TPH-1 and -2 protein levels in hypothalamic tissues were determined by Western blot analysis as described previously (Rahman and Thomas, 2009). Briefly, a protease inhibitor cocktail was added to the homogenization buffer (HEPES buffer containing 25 mM HEPES, 10 mM NaCl, 1 mM EDTA, 1 mM dithioerythritol, pH 7.6) to prevent degradation of TPH proteins during homogenization. The tissue homogenate were centrifuged at 10,000 ×*g* for 15 min at 4°C and the supernatant was used for Western blot analysis. The proteins were solubilized by boiling in SDS loading buffer (0.5 M Tris-HCl, 0.5% Bromophenol Blue, 10% glycerol) and cooled on ice for 5 min. The solubilized protein was resolved on a 10% SDS-PAGE gel, transferred onto a immuno-blot polyvinyl difluoride membrane (PVDF membrane, Bio-Rad, Hercules, CA) and blocked with 5% nonfat milk in TBS-T buffer (50 mM Tris, 100 mM NaCl, 0.1% Tween 20, pH 7.4) for 1 h. Membranes were rinsed with TBS-T buffer and probed with primary rabbit anti-TPHs (dilution: 1:1000) or anti-actin (1:10,000; Novus Biologicals) antibodies overnight at 4°C. Membranes were then washed with TBS-T, and incubated for 2 h with a goat polyclonal to rabbit IgG (HRP) secondary antibody (1: 4000; SouthernBiotech). The protein was visualized by the addition of chemiluminescent substrate (Pierce, Rockford, IL) and photographed on Hyperfilm (Amersham Biosciences). The intensities of TPHs and actin protein bands were estimated by ImageJ software to quantify the relative TPHs protein expression.

### Quantitative real-time PCR (qRT-PCR) analysis of TPH mRNAs

To determine the expression of TPH-1 and TPH-2 mRNAs in croaker hypothalamic, qRT-PCR analyses were performed on total RNA using a one step SYBR Green qRT-PCR method as described previously (Rahman and Thomas, 2012, 2013). Briefly, RNA was extracted from hypothalamic tissues using TRI reagent (Sigma-Aldrich) and treated with DNase (Promega) to prevent genomic DNA contamination. Afterwards the RNA was quantified with a NanoDrop 2000C (Thermo Fisher Scientific) and stored at −80°C until use. qRT-PCR analyses were performed using a Brilliant II SYBR Green QRT-PCR master mix kit (Agilent Technologies, La Jolla, CA) in a 25 μl reaction mixture containing 12.5 μl of 2× SYBR-QRT-PCR master mix, 1 μl of RT/RNase block enzyme mixture, 125 nM of each primer, and 2.5 ng of total RNA. The gene-specific primers used for amplification of croaker TPHs mRNA were as follows: TPH-1 (forward: 5′-GAAGACGTGGGGAGTTGTGT-3′ and reverse: 5′-ACAGTGGAAAACACGGAAGG-3′; GenBank accession number EU730759) and TPH-2 (forward: 5′-ATGTTTGACCCGAAGACGAC-3′ and reverse: 5′-GTGTTCATCTTCCCCAGAGC-3′ primers; EU730760). The thermocycler conditions for amplification were 50°C for 30 min, 95°C for 10 min, and 40 cycles of 95°C for 30 s, 55°C for 1 min and 72°C for 30 s. Dissociation curve analyses were performed immediately after the amplification cycles at 95°C for 15 min, 50°C for 15 s. Each transcript level was normalized on the basis of the amplification of croaker 18S rRNA (primers: forward 5′-AGAAACGGCTACCACATCCA-3′ and reverse 5′-TCCCGAGATCCAACTACGAG-3′; AY866435). The mean of threshold cycle (C*t*) was used for the analysis and the relative expression levels of croaker TPHs mRNA against 18S rRNA were calculated using the 2^−ΔΔ*Ct*^ method (Livak and Schmittgen, 2001).

### Radioenzymatic assay (REA) of TPH activity

TPH activity was measured by REA (Vrana et al., 1993) using [^3^H]-tryptophan tracer and validated for croaker brain as described previously (Khan and Thomas, 2001; Rahman and Thomas, 2009, 2013). Briefly, hypothalamic tissues were sonicated in HEPES buffer (pH 7.6). Twenty five μl of tissue homogenate was added to 25 μl of reaction mixture containing 0.05 mM tryptophan, 50 mM HEPES (pH 7.6), 5 mM DTT, 0.01 mM Fe(NH_4_)_2_(SO_4_)_2_, 0.5 mM 6-MPH_4_, 0.1 mg/ml catalase, and 1 μCi L-[5-^3^H]-tryptophan. The reaction mixture was incubated at 25°C for 20 min in a water bath and stopped by addition of 500 μl of ice-cold 7.5% charcoal in 1 M HCl. The reaction mixture was centrifuged at 14,000 ×*g* for 2 min and the supernatant (200 μl) was added to 3 ml of CytoScint scintillation cocktail (MP Biomedicals, Irvine, CA). The radioactivity was measured by a liquid scintillation counter (Beckman LS 6000SC, Beckman Instruments, Fullerton, CA). The radioactivity derived from each assay was normalized to total protein present in the homogenate as determined by Bradford protein assay. Each assay was done in duplicate and the mean value was expressed as nmol tryptophan converted/mg protein/h.

### High-performance liquid chromatography (HPLC) with electrochemical detection analysis of 5-HT and 5-HTP contents

5-HTP and 5-HT concentrations in hypothalamic tissues were determined by reversed-phase HPLC with an electrochemical detection citation(Waters 464 Electrochemical Detector and Breeze Software, Milford, MA) system (Saligaut et al., 1986) according to Khan and Thomas (1997) with minor modifications. Briefly, the frozen hypothalamic tissue samples were sonicated in 0.5 ml of ice-cold perchloric acid (0.2 M) containing 0.1% cysteine, 0.05% sodium bisulfite, and 0.05% EDTA, and centrifuged at 2500 ×*g* for 10 min. The supernatant was filtered through a 0.22 μm Millex-GV filter and immediately injected (50 μl) into the HPLC apparatus. The chromatographic separations were performed by C18 reverse phase column (4.6 × 150 mm, Waters) using a mobile phase buffer (0.3 M acetic acid, 0.08 M ammonium hydroxide, 0.1 mM EDTA, and 75% methanol) at a flow-rate of 1 ml/min at room temperature. The peaks of 5-HTP and 5-HT were identified by comparing the retention times with those of 5-HTP and 5-HT standards, and the concentrations were calculated from the areas under the peaks. 5-HTP and 5-HT contents were normalized to total protein present in the homogenate as determined by Bradford protein assay and expressed as ng/mg protein.

### Statistical analyses

All of the experimental results were analyzed by One-Way ANOVA followed by Fisher's protected least-significant difference test for multiple comparisons. A *P*-value of less than 0.05 was considered statistically significant. Analyses were performed using Statview (SAS Instituite Inc., Cary, NC) and GraphPad Prism (GraphPad, San Diego, CA) computer software. Data are expressed as mean ± standard error of the mean (s.e.m).

## Results

The specificities of the TPHs antibodies for immunohistochemistry (IHC) detection were confirmed by blocking them with specific TPHs peptide antigens (Supplementary Figure 1). Western blot analyses confirmed the presence of immunoreactive (IR) bands of TPHs proteins of the predicted size around 55–56 kDa as shown previously in croaker hypothalamus (Rahman and Thomas, 2009).

**Figure 1:**
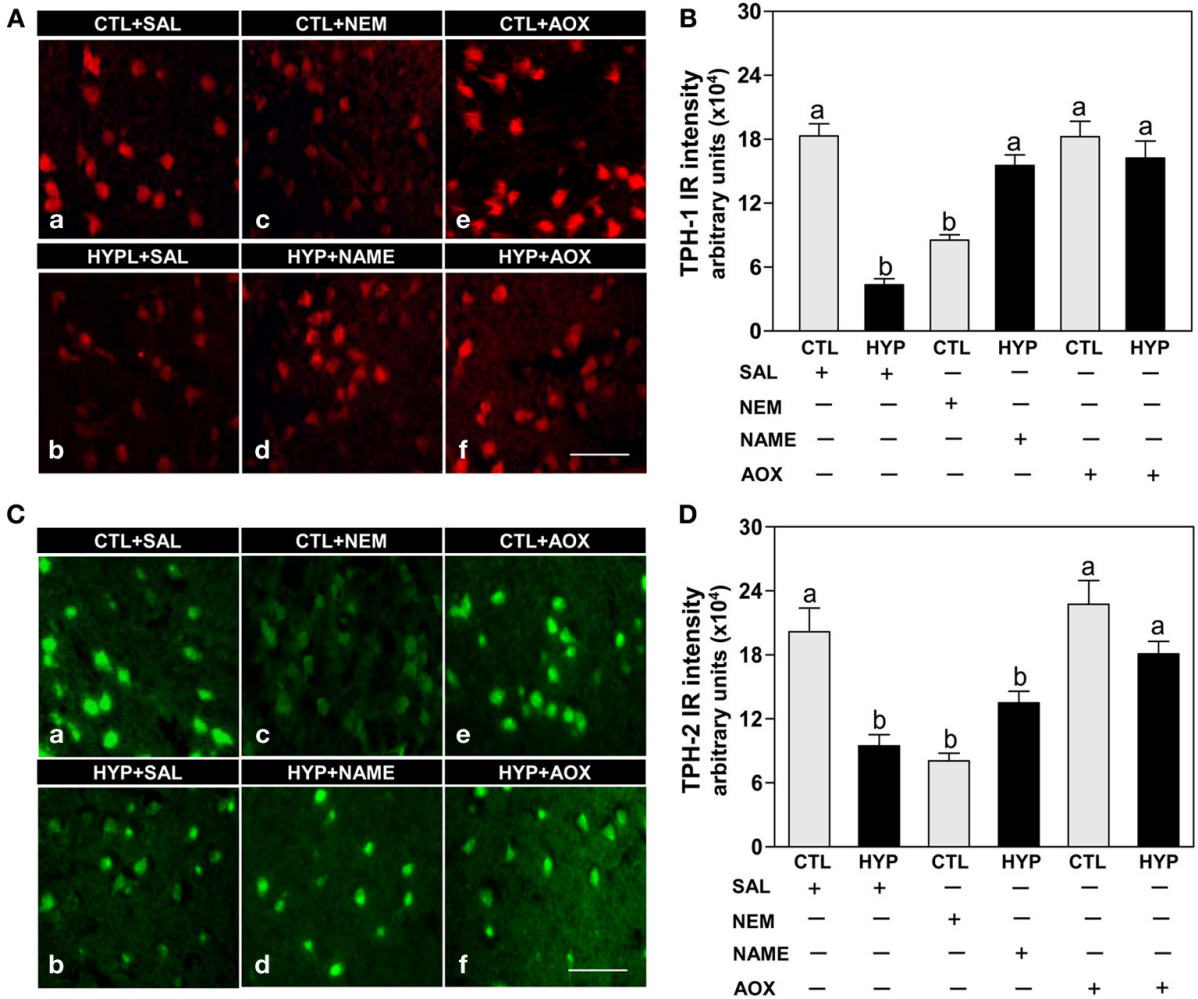
**Interactive effects of hypoxia and pharmacological agents that modulate generation of reactive oxygen and nitrogen species (ROS and RNS) on expression of TPHs proteins in croaker hypothalamic neurons assessed by immunohistochemistry**. Effects of hypoxia (dissolved oxygen, DO: 1.7 mg/L for 4 weeks; note: here and in subsequent figures exposure duration only refers to period fish were exposed to target DO; fish were previously exposed to declining DO for additional 2-day adjustment period) exposure and pharmacological treatments with *N*-ethylmaleimide (NEM, a chemical which covalently modifies sulfhydryl groups in the reactive center of enzymes), *N*ω-nitro-L-arginine methyl ester (NAME, an inhibitor of nitric oxide synthase) and vitamin E (an antioxidant, AOX) on immunohistochemical (IHC) expression and immunoreactive (IR) intensity of TPH-1 **(A,B)** and TPH-2 **(C,D)** neurons in croaker hypothalamus. **(A,C)** Representative IHC micrographs of TPHs neurons in hypothalamic sections from fish after the various treatments. Scale bar = 20 μm. **(B,D)** IR staining intensity of the fluorescent-labeled TPHs neurons assayed by fluorescein filter with Nikon Eclipse microscope, and the IR staining intensity of each neuron estimated by ImageJ software (http://rsb.info.nih.gov/ij/) according to Collins (2007). Each value represents the mean ±s.e.m. (*N* = 25–33 neurons). Significant differences as compared to control (CTL) identified with a multiple range test, Fisher's PLSD, are indicated with different letters (*P* < 0.05). SAL, saline; CTL, control; HYP, hypoxia.

Immunohistochemical results showed strong expression and high intensity of TPH-1 immunoreactive (IR) neurons in hypothalami of fish exposed to normoxic control conditions, whereas weak expression and low intensity of TPH-1 IR neurons were observed in hypoxia-exposed (1.7 mg DO/L for 4 weeks) saline-injected control fish (Figures 1Aa,b,B). Administration of the sulfhydryl modifying agent, NEM (1 μg/g BW/4 days for 4 weeks), markedly reduced TPH-1 neuronal expression and intensity compared to normoxic saline controls (Figures 1Ac,B), whereas treatments with the NOS-inhibitor, NAME, or the antioxidant, vitamin E (1 μg NAME or 1 μg Vit E/g BW/4 days for 4 weeks), restored the hypoxia down-regulation of TPH-1 neuronal expression and intensity (Figures 1Ad,f,B). A similar pattern of treatment effects were observed in TPH-2 neurons in croaker hypothalami (Figures 1C,D).

**Figure 2:**
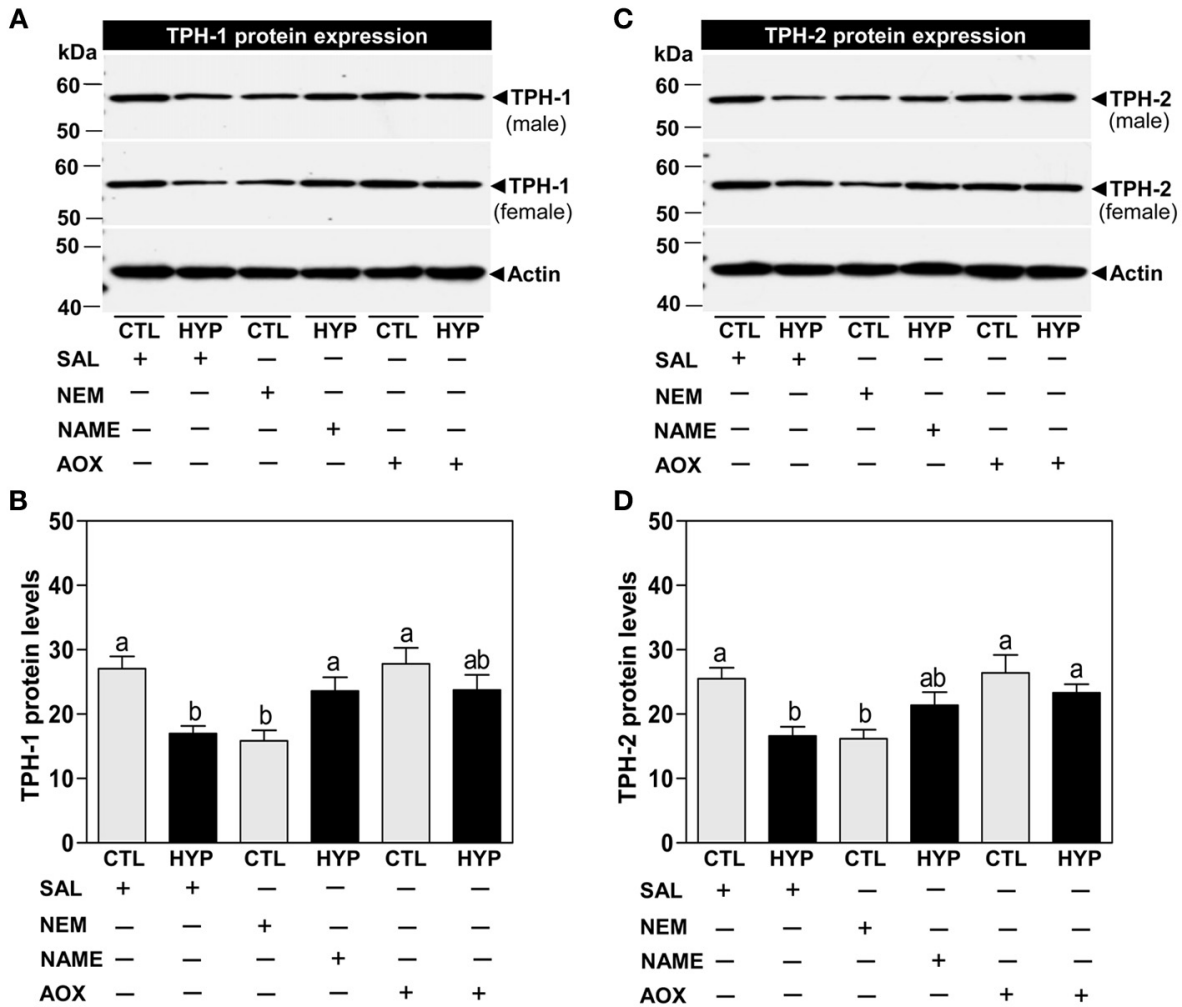
**Interactive effects of hypoxia and pharmacological agents that modulate generation of ROS and RNS on TPHs protein expression determined by Western blot analysis in croaker hypothalamus**. Effects of hypoxia (DO: 1.7 mg/L for 4 weeks) exposure and pharmacological treatments with NEM, NAME, and AOX on immunoreactive (IR) expression and IR intensity of TPH-1 **(A,B)** and TPH-2 **(C,D)** protein in croaker hypothalamus. **(A,C)** Representative Western blot shown for samples from individual male and female fish after the various treatments. **(B,D)** IR protein intensity estimated by ImageJ software. Each bar represents mean ±s.e.m. (*N* = 8, results from both sexes were combined because they were not significantly different). Fisher's PLSD, are indicated with different letters (*P* < 0.05). SAL, saline; CTL, control; HYP, hypoxia.

Immunoblot results showed that the expression of TPH-1 IR signal was significantly reduced (~37%) in hypoxia-exposed fish compared with controls following normalization to actin protein (Figures 2A,B). Treatment with NEM for 4 weeks caused similar decreases in TPH-1 protein expression and levels to those observed after hypoxia exposure, whereas treatments with NAME or Vit E restored TPH-1 protein levels in hypoxia-exposed fish (Figures 2A,B). Similar to the TPH-1 IR signal, hypothalamic TPH-2 protein expression and levels were restored after treatments with NAME or vitamin E in hypoxia-exposed fish (Figures 2C,D).

**Figure 3:**
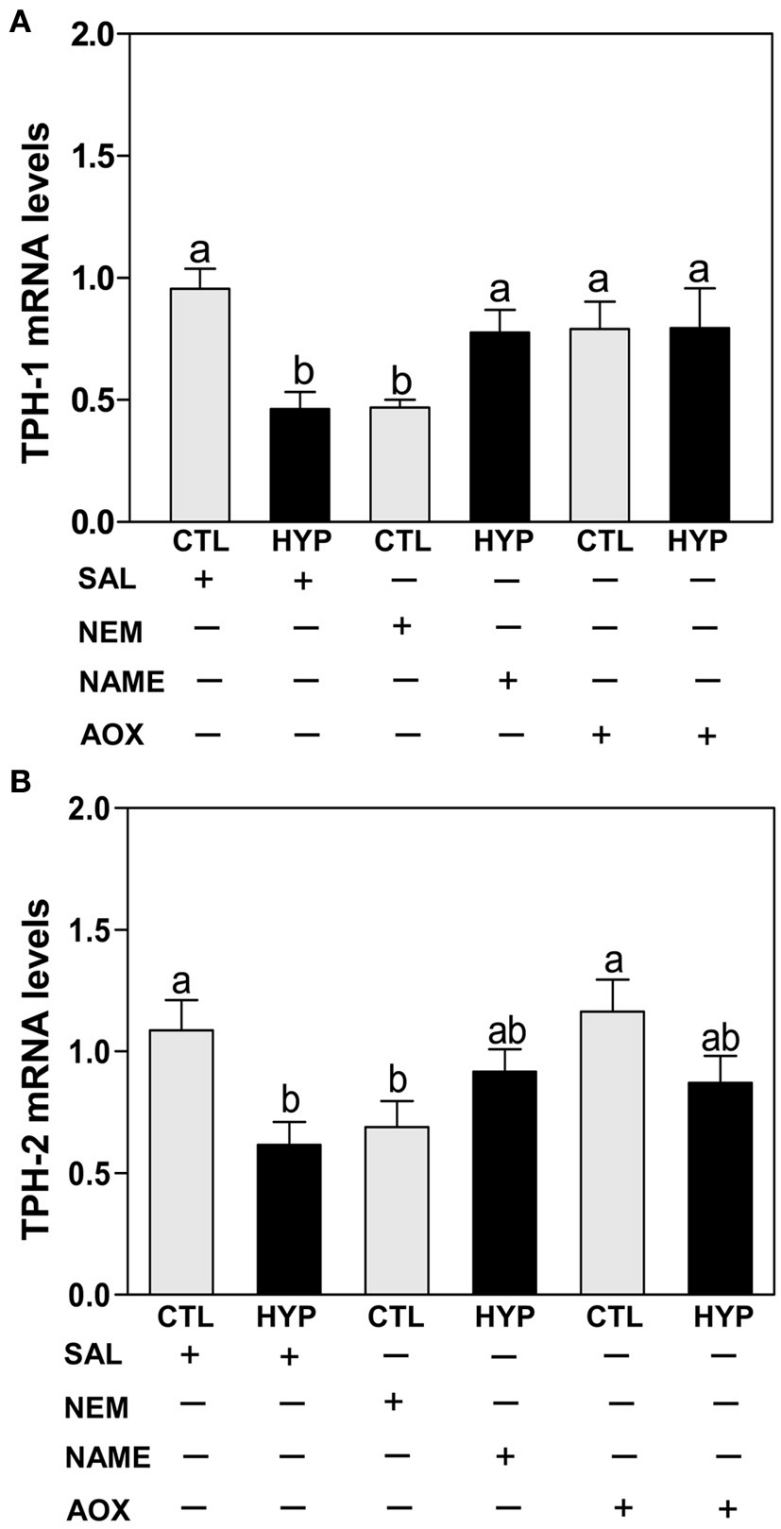
**Interactive effects of hypoxia and pharmacological agents that modulate generation of ROS and RNS on TPHs mRNA expression determined by quantitative real-time PCR in croaker hypothalamus**. Effects of hypoxia (DO: 1.7 mg/L for 4 weeks) exposure and pharmacological treatments with NEM, NAME, and AOX on expression of TPH-1 **(A)** and TPH-2 **(B)** mRNA levels in croaker hypothalamus. Each bar represents mean ±s.e.m. (*N* = 9–12, results from both sexes were combined because they were not significantly different). Fisher's PLSD, are indicated with different letters (*P* < 0.05). SAL, saline; CTL, control; HYP, hypoxia.

Quantitative real-time PCR (qRT-PCR) showed that exposure to hypoxia and treatment with NEM caused comparable decreases in hypothalamic TPH-1 (Figure 3A) and TPH-2 (Figure 3B) mRNA levels compared to normoxic saline controls, whereas treatments with NAME or Vit E completely and partially restored TPH-1 (Figure 3A) and TPH-2 (Figure 3B) mRNA levels, respectively, in hypoxia-exposed fish.

**Figure 4:**
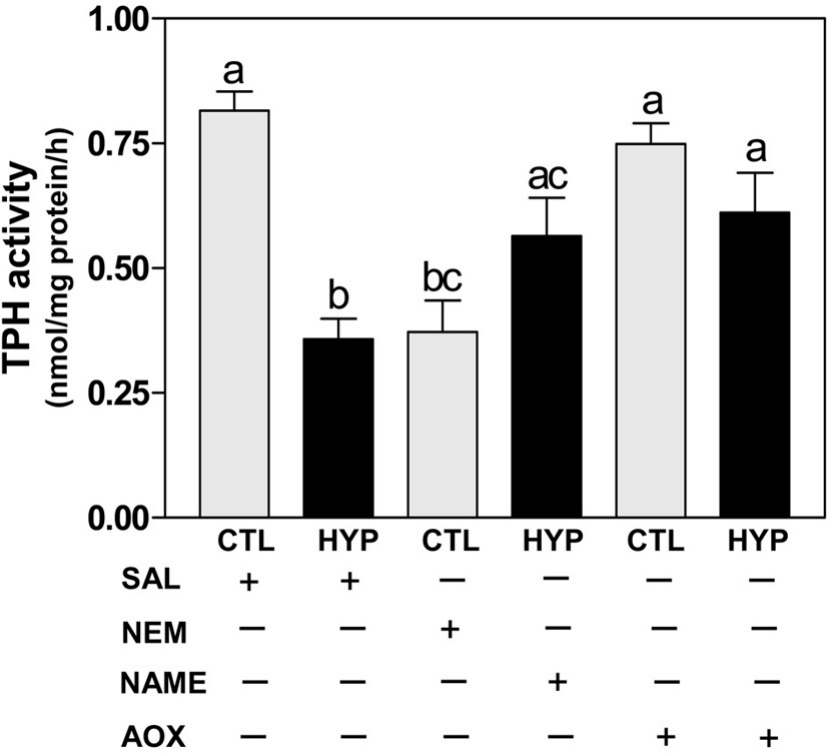
**Interactive effects of hypoxia and pharmacological agents that modulate generation of ROS and RNS on TPH activity determined by radioenzymatic assay in croaker hypothalamus**. Effects of hypoxia (DO: 1.7 mg/L for 4 weeks) exposure and pharmacological treatments with NEM, NAME, and AOX on TPH activity in croaker hypothalamus. Each bar represents mean ±s.e.m. (*N* = 6–8, results from both sexes were combined because they were not significantly different). Fisher's PLSD, are indicated with different letters (*P* < 0.05). SAL, saline; CTL, control; HYP, hypoxia.

The TPH radioenzymatic assay results showed a dramatic decrease (~56%) in hypothalamic TPH activity in hypoxia-exposed fish compared with normoxic saline controls (Figure 4). TPH activity was also significantly decreased by treatment with NEM to a level similar to that observed in the hypoxia-exposed fish, whereas TPH activity was markedly restored (~70% for NAME and ~75% for Vit E treatments) in hypoxia-exposed fish by treatment with NAME or Vit E (Figure 4).

**Figure 5:**
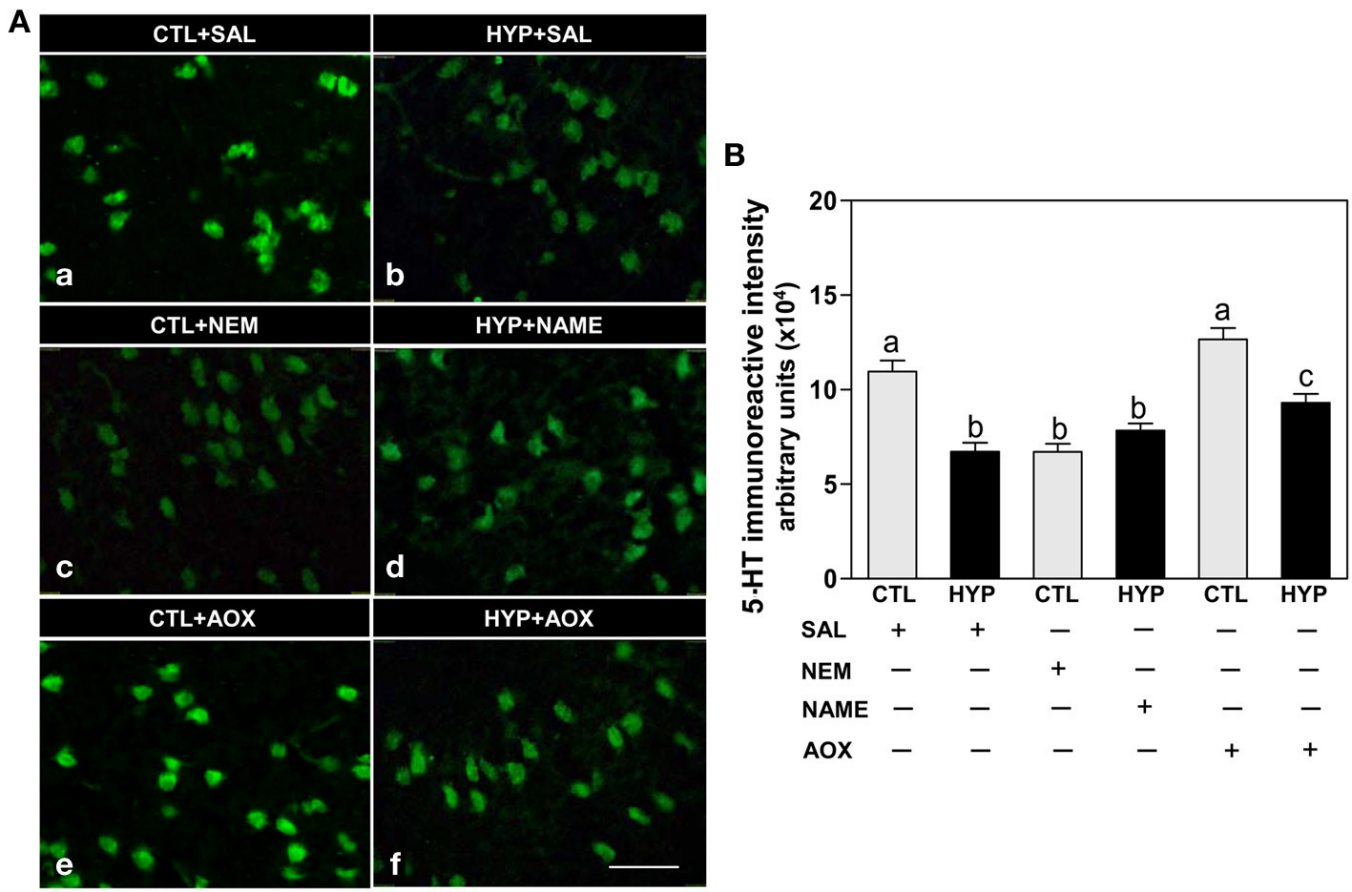
**Interactive effects of hypoxia and pharmacological agents that modulate generation of ROS and RNS on 5-HT content in neurons assessed by immunohistochemistry in croaker hypothalamus**. Effects of hypoxia (DO: 1.7 mg/L for 4 weeks) exposure and pharmacological treatments with NEM, NAME, and AOX on immunohistochemical (IHC) expression and immunoreactive (IR) intensity of 5-HT neurons in croaker hypothalamus. **(A)** Representative IHC micrographs of 5-HT neurons in hypothalamic sections from fish after the various treatments. Scale bar = 20 μm. **(B)** IR staining intensity of the fluorescent-labeled 5-HT neurons assayed by fluorescein filter with Nikon Eclipse microscope, and the IR staining intensity estimated by ImageJ software. Each value represents the mean ±s.e.m. (*N* = 23–26 neurons). Significant differences as compared to control (CTL) identified with a multiple range test, Fisher's PLSD, are indicated with different letters (*P* < 0.05). SAL, saline; CTL, control; HYP, hypoxia.

Immunohistochemical results showed weak expression and low intensity of 5-HT IR in hypothalamic neurons of fish exposed to hypoxia or treatment with NEM compared to normoxic saline controls (Figures 5Aa-c,B), whereas NAME or Vit E treatments partially restored 5-HT IR neuronal expression and intensity in hypoxia-exposed fish (Figures 5A,d,f,B).

**Figure 6:**
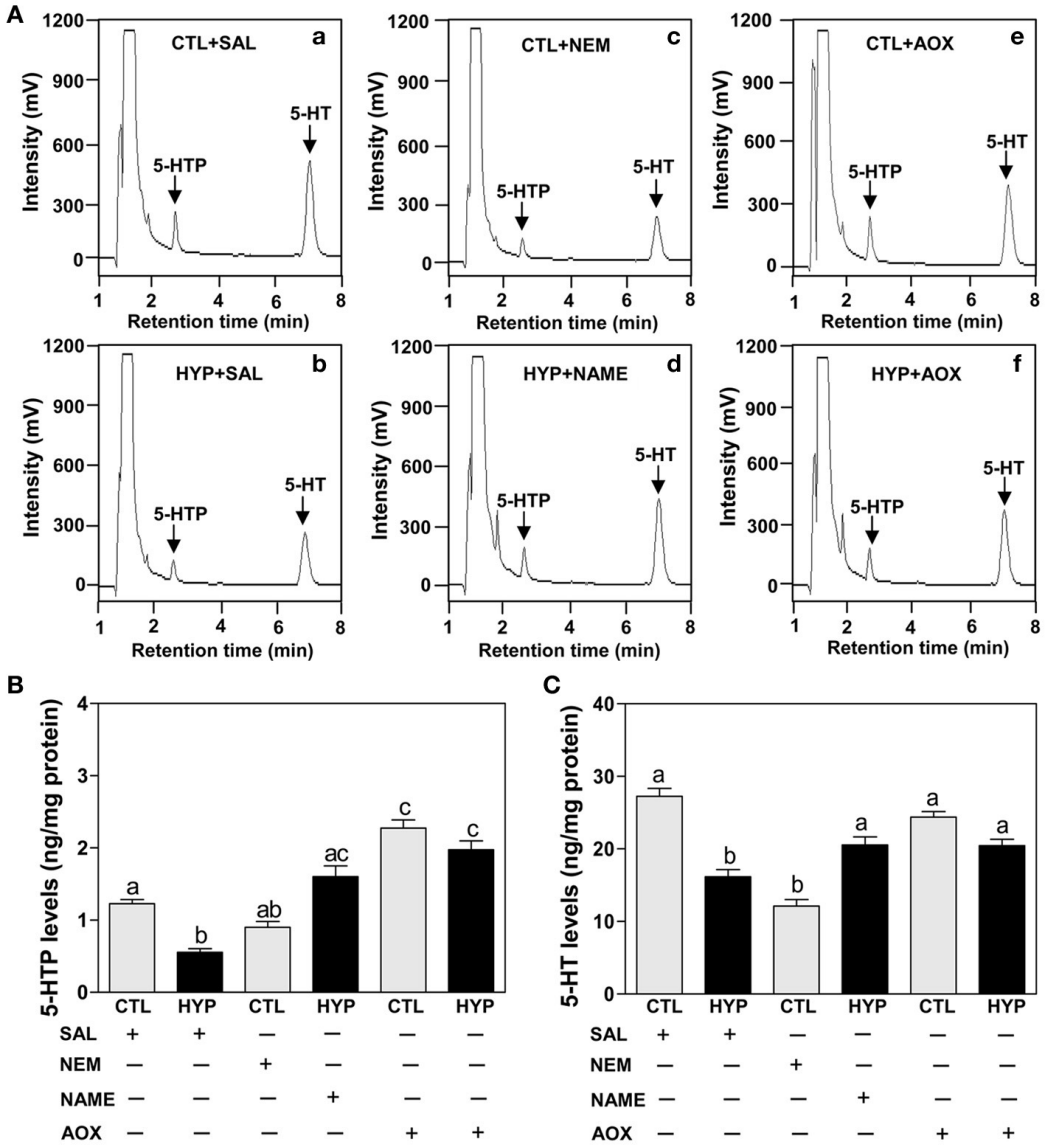
**Interactive effects of hypoxia and pharmacological agents that modulate generation of ROS and RNS on 5-HTP and 5-HT levels determined by HPLC in croaker hypothalamus. (A)** Reversed-phase HPLC chromatograms of 5-HTP and 5-HT showing that hypothalamic 5-HTP and 5-HT contents from normoxic saline control fish are higher than those in hypoxic (DO: 1.7 mg/L for 4 weeks) and NEM treatment group. **(B,C)** Effects of hypoxia (dissolved oxygen, DO: 1.7 mg/L for 4 weeks) exposure and pharmacological treatments with NEM, NAME, and AOX on 5-HTP **(B)** and 5-HT **(C)** levels in croaker hypothalamus. Each value represents the mean ±s.e.m. (*N* = 7–9, results from both sexes were combined because they were not significantly different). Significant differences identified with a multiple range test, Fisher's PLSD, are indicated with different letters (*P* < 0.05). SAL, saline; CTL, control; HYP, hypoxia.

The HPLC results show exposure to hypoxia significantly decreased 5-HTP and 5-HT contents in hypothalamic tissues compared to normoxic saline controls (Figures 6Aa,b,B,C). Injection with NEM caused a moderate decrease in 5-HTP content in normoxic fish (Figures 6Ac,B), whereas NEM treatment caused a marked decrease 5-HT content similar to that observed in hypoxia-exposed fish (Figures 6Ac,C). The hypoxia-induced decreases in 5-HTP and 5-HT contents were restored by treatments with NAME or Vit E in hypoxia-exposed fish (Figures 6Ad,f,B,C).

**Figure 7:**
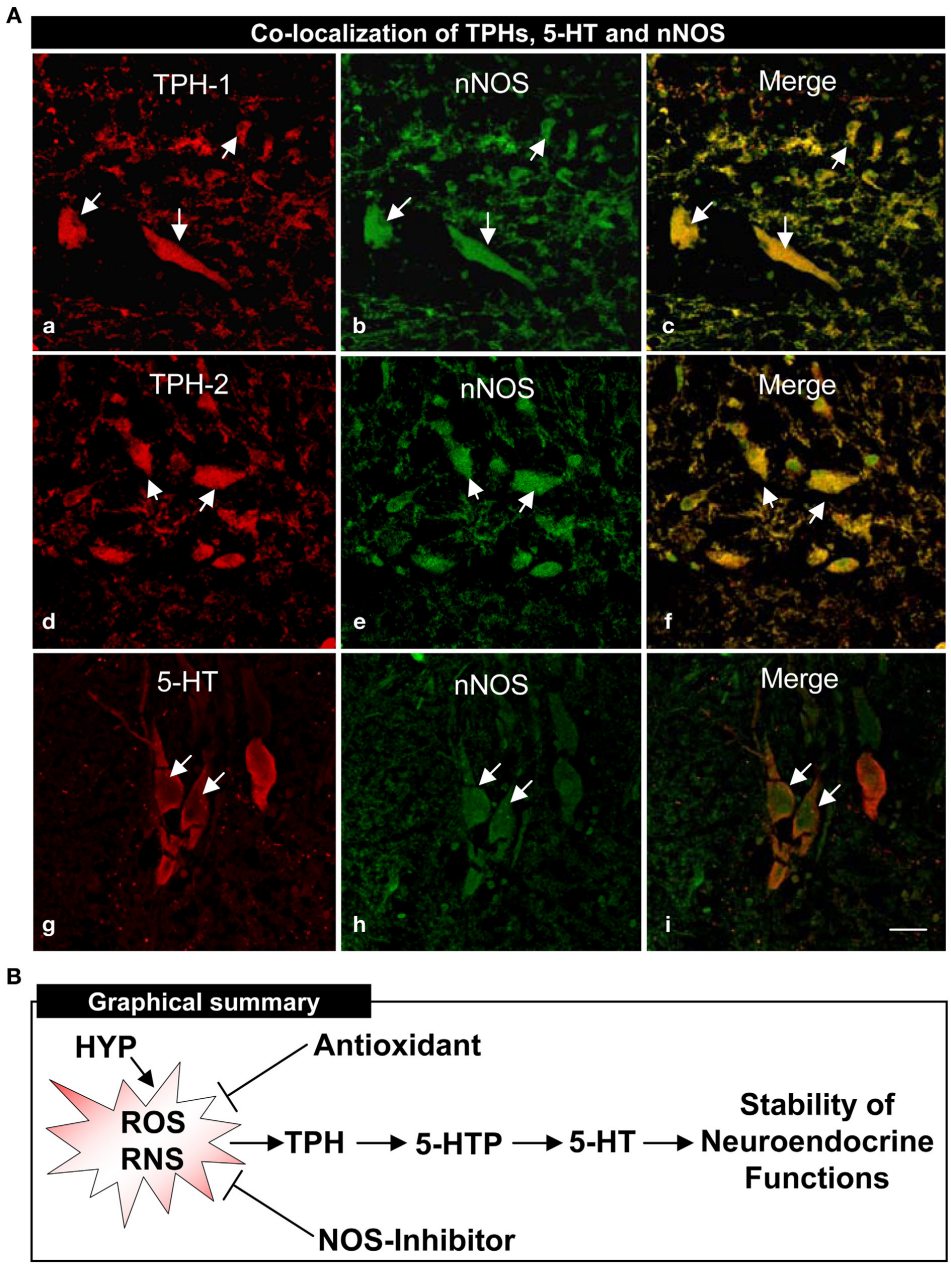
**Co-localization of TPHs and 5-HT neurons on neuronal nitric oxide synthase (nNOS) determined by double-immunofluorescence in croaker hypothalamus and proposed model of their interactions (A)**. Immunohistochemical co-localization of TPH-1 **(a–c)**, TPH-2 **(d–f)** and 5-HT **(g–i)** neurons with nNOS neurons. Scale bar = 5 μm. Arrows indicate neuronal expression of TPH-1, TPH-2, 5-HT, nNOS and their co-expression. **(B)** Proposed model of hypoxia down-regulation of TPH and 5-HT regulation through the generation of reactive oxygen and nitrogen species (ROS and RNS) in croaker hypothalamus. The antioxidant (Vitamin E) and nitric oxide synthase (NOS)-inhibitor block hypoxia-induced down-regulation of TPH activity, 5-HTP, and 5-HT contents and restore the neuroendocrine functions.

An anatomical basis for the interactions between TPHs or 5-HT neurons and nNOS in croaker hypothalamic was investigated by double-immunofluorescence assay. Immunohistochemical results showed that TPH-1, TPH-2, and 5-HT are co-expressed with nNOS in neurons in croaker hypothalami as seen in the merged images (Figure 7A).

## Discussion

In this report, several potential components of the mechanism by which hypoxia down-regulates TPHs (TPH-1 and TPH-2) and 5-HT functions in the Atlantic croaker hypothalamus were identified. The observation that repeated injections with vitamin E (Vit E) substantially restored hypothalamic TPHs expression and 5-HT concentrations suggests that increasing the antioxidant status can prevent hypoxia-induced down-regulation of the serotonergic system. In addition, the finding that systemic administration of NOS-inhibitor, NAME, partially reversed the hypoxia down-regulation of TPHs and 5-HT neuronal expression suggests that the hypoxia effects on the serotonergic system are mediated through the enzyme, nNOS. Moreover, the demonstration that TPH and nNOS proteins are co-localized in croaker hypothalamic neurons provides the neuroanotomical basis for close interactions between these two enzymes. Reactive oxygen and nitrogen species (ROS and RNS) are common denominators in all these studies because they are generated during hypoxia and through nNOS and the antioxidant Vit E can block their generation. On the basis of these results we propose that hypoxia causes down-regulation of TPH mRNA expression through the generation of ROS and RNS resulting in reduced TPH protein synthesis and activity and declines in 5-HT levels and neuroendocrine functions (Figure 7B). The mechanism(s) by which ROS and RNS alter TPH mRNA expression remains unclear but may involve upregulation of hypoxia-inducible factor-α (HIF-α, an oxygen-sensitive transcription factor) and down-regulation of aromatase (Rahman et al., 2011; Rahman and Thomas, 2013). To our knowledge, this is the first evidence that NOS activity and antioxidant status influence neuronal TPH and 5-HT functions in aquatic vertebrates during hypoxic stress.

The present results showing administration of NAME, an inhibitor of NOS partially restored TPHs (TPH-1 and TPH-2) neuronal, protein and mRNA expressions, TPH activity and 5-HT concentrations in the hypothalamus clearly implicate nNOS activity and nitric oxide (NO) generation as intermediaries in these hypoxia-induced effects. In agreement with these findings, *in vivo* administration of NO inhibitors has been shown to increase neuronal contents of TPH and 5-HT in rat raphe nuclei and 5-HT release from the hippocampus and hypothalamus under normoxic conditions (Kaehler et al., 1999; Wegener et al., 2000; Park et al., 2004). Conversely, administration of *S*-nitroso-*N*-acetypenicillamine (SNAP, a NO-donor) markedly decreases 5-HT contents in the hypothalamus, frontal cortex and raphe nuclei of rat brains under normoxic conditions (Kaehler et al., 1999; Smith and Whitton, 2000), suggesting that expression of both TPH and 5-HT are down-regulated by NO. Several mammalian *in vitro* studies have also been shown that hypoxia increases cellular NO levels, and that excess NO interacts with superoxide radical (O^−^_2_, a potent ROS) to generate peroxynitrite (ONOO^−^, a highly diffusible RNS; Beckman and Koppenol, 1996) resulting in oxidative damage in a wide array of cells including neurons (Encinas et al., 2003; Maiti et al., 2010). Consistent with these *in vitro* studies, Maulik et al. (1998) and Mishra et al. (2006) demonstrated that hypoxia increases free radicals and nNOS protein levels in guinea pig and piglets brain tissues *in vivo*. We have recently demonstrated that hypoxia markedly increases plasma NO metabolites (NOx, nitrite, and nitrate), NOS mRNA and protein levels in croaker liver and brain tissues (Rahman and Thomas, 2011; Rahman and Thomas, unpublished observation). These results are consistent with the findings of other teleost studies showing that hypoxia significantly increases plasma NOx concentrations and neural NOS mRNA levels in trout (McNeill and Perry, 2006) and protein carbonyl contents, an indirect measure of ROS (Berlett and Stadtman, 1997), in grouper liver (Yu et al., 2008). Taken together these studies in fish and mammals suggest that hypoxia causes elevated NO-free radical production, such as ONOO^−^ through an increase in NOS activity that can result in inhibition of TPH expression and activity as well as impairment of serotonergic functions (Kuhn and Geddes, 1999, 2000).

Another significant finding in this study is that Vit E, a potent peroxyl radical scavenger (Traber and Atkinson, 2007), treatment fully restored hypothalamic TPHs neuronal, protein and mRNA expressions, and TPH activity in hypothalamus of hypoxia-exposed fish to those observed in normoxic fish. As predicted, Vit E treatment also restored the immunoreactive 5-HT expression in neurons and the contents of 5-HTP and 5-HT in the hypothalami of hypoxia-exposed fish. The results are consistent with those in an *in vivo* study in rats showing that hypoxia markedly decreases monoaminergic neurotransmitters such as norepinephrine, dopamine and 5-HT levels in cerebral cortex, hippocampus and striatum, and that the hypoxia-induced decrease in neurotransmitter levels is restored by treatment with Vit E (Yan et al., 2003). Numerous mammalian *in vitro* studies have shown that antioxidants such as Vit C and Vit E decrease cellular oxidative stress and prevent neuronal cell death during hypoxic stress (Chow, 1991; Chow et al., 1999; Yamagata et al., 2010; Traber and Stevens, 2011). Using a primary neuronal culture, Lièvre et al. (2000) showed that Vit E treatment prevents hypoxia-induced apoptosis in rat forebrain neurons. Similarly, Tagami et al. (1998) demonstrated that during exposure to hypoxic conditions, Vit E directly reacts with free radicals to prevent apoptosis in rat cortical neurons. Vit E significantly increases the intracellular antioxidant levels and decreases hydroxyl radical (OH^•^, highly reactive ROS) and lipid peroxidation levels in cultured rat neuronal cells under normoxic conditions (Zhang et al., 2004). Moreover, during hypoxia exposure, Vit E prevents hydrogen peroxide (H_2_O_2_, a potent ROS) production in cultured human endothelial cells (Martin et al., 1996). We have recently observed that during hypoxia exposure, Vit E attenuates O^−^_2_ production and NOS protein and mRNA levels in croaker liver and brain tissues (Rahman and Thomas, 2011, 2012; Rahman and Thomas, unpublished observation). Importantly, the present results show that the NOS is distinctly co-expressed with TPHs and 5-HT in neurons in croaker hypothalami which is consistent with mammalian studies showing that both TPH and 5-HT neurons are remarkably co-localized with NOS neurons in raphe nuclei (Lopez et al., 2005; Lu et al., 2010). Collectively, our findings together with other tetrapods results suggest that NOS is a mediator of hypoxia-induced down-regulation of TPH and 5-HT function in croaker hypothalamus through it's upregulation of NO production that can be blocked by Vit E.

There are several potential molecular mechanisms of TPH regulation by hypoxia, and by Vit E and ROS or RNS during hypoxia exposure. The available evidence suggests that hypoxia probably down-regulates TPH transcripts through an O_2_-dependent mechanism. The O_2_-sensitive transcription factor HIF-α, a gene that regulates the expression of numerous genes involved in adaptation to hypoxia (Semenza, 2001; Nikinmaa and Ress, 2005), is likely a key transcriptional regulator of TPH mRNA. Mammalian *in vitro* studies have shown that hypoxia induces cellular ROS levels and that elevated ROS levels leads to increases in HIF-α mRNA and protein levels (Chandel et al., 2000; Pouysségur and Mechta-Grigoriou, 2006). On the contrary, antioxidants prevent hypoxia-induced ROS generation, and HIF-α mRNA and protein accumulation in cultured mammalian cells (Sanjuán-Pla et al., 2005). We have recently shown that hypoxia increases O^−^_2_ generation, HIF-1α mRNA expression, and HIF-2α and endothelial NOS (eNOS) protein levels in croaker liver tissues and treatment with Vit E attenuates all of these hypoxia-induced increases (Rahman and Thomas, 2011, 2012). Vit E is a prototypical scavenger of ROS and RNS, and eliminates the hypoxia-induced activation of HIF-α. Therefore, our results support the idea that the decline of TPHs transcript levels observed during hypoxia exposure could involve the activation of HIF-α that may interact with a specific hypoxia-response element in the enhancing region on the TPH promoter causing a decrease in the rate of TPH transcription (Semenza et al., 1996). In addition, the enzymatic activity of TPH may be directly affected by a reduction of O_2_ concentrations because it uses O_2_ as a substrate with the cofactor tetrahydrobiopterin (BH4) required for catalytic conversion of tryptophan to 5-HTP (Cash, 1998). Additional research will be required to determine which of these potential mechanisms have a major influence on serotoneric functions during hypoxia stress.

Hypoxia could also potentially regulate TPH functions through a post-transcriptional mechanism(s). The finding that NEM, a sulfhydryl (SH) blocker that alkylates endogenous SH groups on proteins and peptides, mimics the effects of hypoxia on TPH activity and 5-HT are consistent with such a mechanism. NEM alkylates glutathione (GSH, a major antioxidant present in cells and tissues; Schulz et al., 2000) and cysteine, an amino acid which serves an important role to protect many proteins against oxidative damage (Requejo et al., 2010). The present results are consistent with results of *in vivo* studies showing that NEM treatment drastically suppresses 5-HT content (~43%) in normoxic rat brains (Garzon et al., 1990). *In vitro* studies have also shown that treatment with SH alkalizing agents such as NEM and iodoacetate, suppresses TPH and 5-HT transporter activities in rat brain tissue under normoxic conditions (Kuhn et al., 1980; Wolfel et al., 1989; Wolf and Kuhn, 1992). On the contrary, treatment with various SH agents such as GSH increase TPH activity in rat brain tissues *in vitro* (Kuhn and Arthur, 1997a,b; Hussain and Mitra, 2004), suggesting that the catalytic activity and post-translational modifications of TPH are dependent on endogenous SH residues (Kuhn et al., 1980). We have no direct evidence in support of this suggestion; however, Kuhn and Arthur (1996, 1997a,b) demonstrated that treatments with various RNS and ROS such as NO and H_2_O_2_, inactivate TPH activity via SH oxidation in rat brain extracts *in vitro*. Similarly, Hussain and Mitra (2004) have shown that H_2_O_2_ and exogenous O^−^_2_ suppress TPH activity in rat brain tissues *in vitro*. Interestingly, O^−^_2_ directly reacts with NO to produce ONOO^−^ which inactivates TPH activity by SH oxidation in rat brain tissues (Kuhn and Geddes, 1999). Together these findings suggest that both ROS and RNS are directly involved in inactivation of TPH (Kuhn and Geddes, 2000), and thus this oxygen-liable enzyme could lose its catalytic activity and post-translational modifications when SH residues are decreased in neural tissues during hypoxic stress. There is considerable evidence that exposure to various stresses such as cold, heat, and hypoxia, lead to increased ROS and RNS levels, resulting in decreases in the total SH residues in blood, brain and hepatic tissues in vertebrates (Beck and Linkenheimer, 1952; Bartlett and Register, 1953; Wideman and Domanska-Janik, 1974; Iyer et al., 1985; Farooqui, 2012).

## Conclusions

The results of the present study indicate that during hypoxic stress, administration of a NOS-inhibitor or Vit E leads to restoration of TPH expression and activity and 5-HT content to normal levels in the croaker hypothalamus. This is the first evidence in an aquatic vertebrate for an involvement of NOS in hypoxia down-regulation of TPH and 5-HT and its reversal by an antioxidant. Essentially, Vit E functions as an antioxidant by scavenging excessive free radicals produced during oxidative stress (Traber and Stevens, 2011). Hypoxia also causes down-regulation of reproductive neuroendocrine function in the croaker hypothalamus (Thomas et al., 2007; Thomas and Rahman, 2012). Current studies are investigating whether Vit E exerts a protective effect on hypothalamic neuronal systems related to reproduction during hypoxia stress.

### Conflict of interest statement

The authors declare that the research was conducted in the absence of any commercial or financial relationships that could be construed as a potential conflict of interest.

## Abbreviations

TPH: tryptophan hydroxylase
5-HT: 5-hydroxytryptamine
5-HTP: 5-hydroxytryptophan
NOS: nitric oxide synthase
NO: nitric oxide
ROS: reactive oxygen species
RNS: reactive nitrogen species
DO: dissolved oxygen
IR: immunoreactive
REA: radioenzymatic assay
HPLC: high-performance liquid chromatography
NEM: *N*-ethylmaleimide
NAME: *N*ω-nitro-L-arginine methyl ester
Vit E: vitamin E
AOX: antioxidant.
