# Supplementary material for: Restoration of tryptophan hydroxylase functions and serotonin content in the Atlantic croaker hypothalamus by antioxidant treatment during hypoxic stress
Source: Front Neurosci. 2014 May 30;8:130. doi: 10.3389/fnins.2014.00130 (PMC4038761; doi:10.3389/fnins.2014.00130)
Supplement: Supplementary file 1 [file DataSheet1.PDF]

Supplementary Figure 1

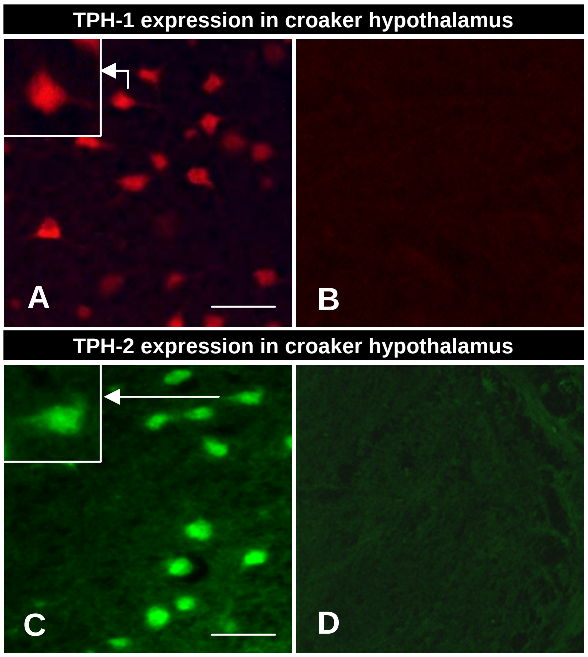

Supplementary Figure 1. Single-labeled immunohistochemistry results showed positive and strong expression of TPH-1 (A) and TPH-2 neurons (C). Immunohistochemical reaction blocked by co-incubation of TPH-1 (B) and TPH-2 (D) antibodies with specific peptide antigens. Scale bar= 20 µm.
